# Evaluating the Readability of Pediatric Neurocutaneous Syndromes–Related Patient Education Material Created by a Custom GPT With Retrieval Augmentation

**DOI:** 10.2196/59054

**Published:** 2025-07-16

**Authors:** Nneka Ede, Robyn Okereke

**Affiliations:** 1 Department of Biomedical Engineering, Cockrell School of Engineering, The University of Texas at Austin, 1500 Red River Street, Austin, TX, 78701, United States, +15124955555; 2 Dell Medical School, The University of Texas at Austin, Austin, TX, United States; 3 Department of Dermatology, Oregon Health and Sciences University, Portland, OR, United States

**Keywords:** ChatGPT, large language model, LLMs, natural language processing, NLP, machine learning, artificial intelligence, generative AI, application programming interface, API, OpenAI, neurocutaneous syndromes, cutaneous, skin, dermatology, patient education, educational, GPT assistant, custom GPT, readability, answer, response, health education

## Abstract

In our study, we developed a GPT assistant with a custom knowledge base for neurocutaneous diseases, tested its ability to answer common patient questions, and showed that a GPT using retrieval augmentation generation can improve the readability of patient educational material without being prompted for a specific reading level.

## Introduction

Children with rare diseases and their families often face the challenge of understanding information regarding such diseases, and educational material is often written above the American Medical Association’s recommended sixth-grade level [1,2]. GPTs can create patient education materials, but their readability often exceeds readers’ comprehension levels [3-5]. GPT assistants are custom GPTs that can use retrieval augmentation generation (RAG) to access specific knowledge [6]. This study aims to evaluate a GPT assistant’s ability to provide readable patient information on pediatric neurocutaneous syndromes in comparison to ChatGPT-4.

## Methods

**Figure 1.**
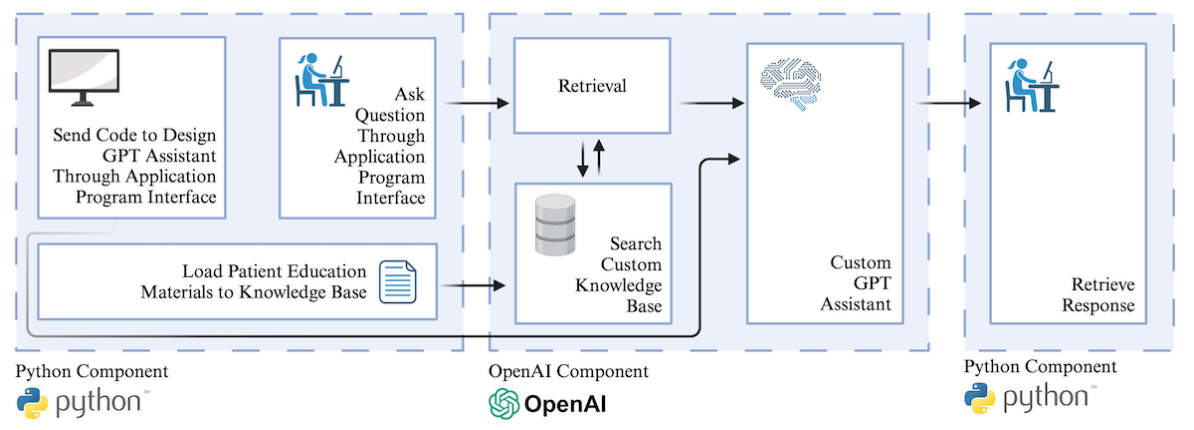
Flow diagram of the creation of the GPT assistant and how it functions. This figure was created in BioRender [7].

A GPT assistant was developed by using Python and OpenAI’s application program interface (API; Figure 1). It was not programmed to answer questions at a specific reading level. Clinician and patient educational materials on four neurocutaneous diseases—tuberous sclerosis complex, neurofibromatosis type 1, neurofibromatosis type 2, and Sturge-Weber syndrome—were integrated into the configuration, with readability ranging from the eighth-grade level to the collegiate level, including sources like UpToDate and Johns Hopkins Medicine.

Five frequently asked patient and caregiver questions surrounding etiology, diagnosis, and management for each of the four diseases were asked to the GPT assistant, with and without a prompt for a response at a sixth-grade reading level (Multimedia Appendix 1). This process was repeated in ChatGPT-4. To minimize overoptimization of the models as questions were asked, no data were cached between API requests, and chat history and training were disabled. Readability was assessed by averaging the following eight readability formulas: Automated Readability Index, Flesch Reading Ease Formula, Gunning Fog Index, Flesch-Kincaid Grade Level Formula, Coleman-Liau Index, SMOG (Simple Measure of Gobbledygook) Index, Linsear Write Formula, and FORCAST Readability Formula (Multimedia Appendix 2) [8]. Two-tailed *t* tests and an ANOVA were used for comparison. Response accuracy was assessed via the OpenFactCheck Python package [9] and then confirmed by the authors (Multimedia Appendix 3).

## Results

The overall average reading level of information generated without any specific prompting for a reading level was 11.4 (SD 2.04) for the custom GPT assistant and 15.41 (SD 2.0) for ChatGPT-4 (Table 1), revealing that the use of a GPT assistant with a knowledge base of patient educational material improved readability by approximately 4 reading levels (*t*_35_=−6.02; *P*<.001). When prompted to answer questions at a sixth-grade reading level, the custom GPT assistant and ChatGPT-4 had average reading levels of 8.8 (SD 0.83) and 9.5 (SD 1.28), respectively, revealing a 0.7 difference in reading level (*t*_38_=−2.05; *P*=.047). The combined use of a GPT assistant and reading level prompt resulted in the best performance (*F*_3,73_=61.74; *P*<.001; Multimedia Appendix 4).

**Table 1.** Average of readability scores for responses generated by the custom GPT assistant without a prompt for reading level, by ChatGPT-4 without a prompt for reading level, by the custom GPT assistant with a prompt for a sixth-grade reading level, and by ChatGPT-4 with a prompt for a sixth-grade reading level. The average reading grade level is an average of 8 common readability formulas.

Metrics	Custom GPT assistant	ChatGPT-4	Custom GPT assistant + prompted reading level	ChatGPT-4 + prompted reading level
Average reading grade level, mean (SD)	11.40 (2.04)	15.41 (2.0)	8.80 (0.83)	9.50 (1.28)
Automated Readability Index, mean (SD)	11.68 (2.54)	16.60 (2.45)	9.30 (1.00)	10.04 (1.62)
Flesch Reading Ease, mean (SD)	49.95 (14.84)	23.41 (12.47)	74.65 (5.39)	69.70 (7.34)
Gunning Fog Index, mean (SD)	13.93 (2.51)	18.41 (2.57)	10.23 (1.05)	10.84 (1.72)
Flesch-Kincaid Grade Level, mean (SD)	10.79 (2.32)	15.32 (2.20)	7.56 (0.98)	8.24 (1.45)
Coleman-Liau Index, mean (SD)	11.70 (2.63)	16.07 (2.14)	8.21 (0.94)	9.21 (1.29)
SMOG^a^ Index, mean (SD)	10.09 (1.84)	13.37 (1.77)	6.69 (0.92)	7.38 (1.37)
Linsear Write score, mean (SD)	11.88 (2.68)	16.09 (2.73)	10.35 (1.49)	10.83 (2.07)
FORCAST readability	10.85 (1.18)	12.10 (0.74)	8.99 (0.46)	9.28 (0.76)

a SMOG: Simple Measure of Gobbledygook.

## Discussion

The GPT assistant provided more readable responses about pediatric neurocutaneous diseases than ChatGPT-4 when no reading level was specified and when a reading level was prompted. Using the GPT assistant with a reading level prompt achieved the best results, suggesting that when a GPT assistant accesses educational materials with a variety of reading levels, readability improves. However, specifying a reading level in ChatGPT-4 resulted in better performance than the GPT assistant without a reading level prompt. Furthermore, there is only a small difference in reading level between models when a comprehension level is prompted, indicating that this action enhances readability, though this is not always intuitive for users. GPT assistants provide another avenue for improving readability, with or without a reading level prompt.

This study also indicated that the caliber of data used when designing a GPT directly influences model results. Poor data quality affects machine learning models’ performance. In the context of readability, poor quality equates to resources with high reading levels. RAG in a GPT assistant allows access to materials with lower reading levels, thereby improving responses without the need for specific prompts. Recent research has determined that RAG improves patient information accuracy and reduces GPT hallucinations; our results show that it can also improve readability [10,11]. If all documents were at a sixth-grade level, readability may improve further; however, more research is needed to determine this.

GPT assistants have the potential to give pediatric dermatology patients and their families another modality for learning and asking questions about the conditions they face—one that is more understandable than ChatGPT alone. Furthermore, GPT assistants may enable clinicians to fine-tune information produced by a GPT specifically for their patient population. GPT assistants with a knowledge base incorporating easy-to-read material can better aid physicians in providing patient- and caregiver-level information, with or without a specific reading level prompt, when compared to ChatGPT-4 alone. A limitation of this study is the limited number of questions assessed. However, this study provides a foundation for larger-scale future research.

## Supplementary material

10.2196/59054 Multimedia Appendix 1 Prompts input into the GPT assistant and ChatGPT.

10.2196/59054 Multimedia Appendix 2 Readability formula definitions.

10.2196/59054 Multimedia Appendix 3 Supplemental methods and results for response accuracy.

10.2196/59054 Multimedia Appendix 4 ANOVA results.

## Abbreviations

API: application program interface
RAG: retrieval augmentation generation
SMOG: Simple Measure of Gobbledygook
