# Supplementary material for: Evaluating the Readability of Pediatric Neurocutaneous Syndromes–Related Patient Education Material Created by a Custom GPT With Retrieval Augmentation
Source: JMIR Dermatol. 2025 Jul 16;8:e59054. doi: 10.2196/59054 (PMC12286582; doi:10.2196/59054)
Supplement: Multimedia Appendix 1 [file derma-v8-e59054-s001.docx]

List of questions input into both the custom GPT Assistant and ChatGPT-4

4 Neurocutaneous Diseases

1. Tuberous Sclerosis Complex
2. Neurofibromatosis Type 1
3. Neurofibromatosis Type 2
4. Sturge-Weber Syndrome

Questions (for each blank input the diseases [1 - 4]):

1. Can you explain the signs and symptoms of __?
2. Can you explain how physicians diagnose __?
3. Can you explain how physicians manage __?
4. Can you explain what causes __?
5. Can you explain the outcomes of __?
6. Can you explain the signs and symptoms of __ at a 6th-grade reading level?
7. Can you explain how physicians diagnose __ at a 6th-grade reading level?
8. Can you explain how physicians manage __ at a 6th-grade reading level?
9. Can you explain what causes __ at a 6th-grade reading level?
10. Can you explain the outcomes of __ at a 6th-grade reading level?
