# Supplementary material for: Evaluating the Readability of Pediatric Neurocutaneous Syndromes–Related Patient Education Material Created by a Custom GPT With Retrieval Augmentation
Source: JMIR Dermatol. 2025 Jul 16;8:e59054. doi: 10.2196/59054 (PMC12286582; doi:10.2196/59054)
Supplement: Multimedia Appendix 2 [file derma-v8-e59054-s002.docx]

Readability Formula Definitions.

| Automated Readability Index | Designed to estimate the readability of technical documents based on the number of characters and the number of words per sentence. |
| --- | --- |
| Flesch Reading Ease | Designed to estimate the reading level based on the number of syllables in each word. |
| Gunning Fog Index | Designed to estimate readability based on average sentence length and number of complex words, typically used for digital content, articles, and textbooks. |
| Flesch-Kincaid Grade Level | Designed to estimate the reading level by calculating sentence length and word diversity. |
| Coleman-Liau Index | Designed to estimate the reading level by focusing on the average number of letters and sentences. |
| SMOG (Simple Measure of Gobbledygook) Index | Designed to estimate the readability of long text, focusing on polysyllabic words. |
| Linsear Write Readability Formula | Designed to estimate the reading level of text based on sentence length and 3+ syllable words. |
| FORCAST Readability Formula | Designed to estimate the reading level of specialized texts, including technical material and procedural writings, based on one-syllable words. |
