# Supplementary material for: Evaluating the Readability of Pediatric Neurocutaneous Syndromes–Related Patient Education Material Created by a Custom GPT With Retrieval Augmentation
Source: JMIR Dermatol. 2025 Jul 16;8:e59054. doi: 10.2196/59054 (PMC12286582; doi:10.2196/59054)
Supplement: Multimedia Appendix 3 [file derma-v8-e59054-s003.docx]

Supplemental Methods and Results for Response Accuracy

Methods

Accuracy of GPT responses was assessed in Python using the package OpenFactCheck [8], which grades the accuracy of each sentence/fact with a generated response. The average number of facts per response was 17. The factuality of the generated response was determined to be overall “true” if all facts were true, for an accuracy of 100%. If any fact within a response contained false information, the overall factuality was determined to be “false”, and the accuracy it was calculated as the percentage of correct statements within that response. Accuracy of each fact was then verified manually by the authors.

Results

Out of the 80 total responses, 74 were completely accurate (92.5%). Across the four GPT models, all performed with high accuracy, >99% (Table 2). There were no entirely fabricated statements.

*Table S1. Accuracy of Responses*

| *Model* | *Average Accuracy* |
| --- | --- |
| Custom GPT Assistant | 99.45% |
| ChatGPT-4 | 99.40% |
| Custom GPT Assistant + Prompted Reading Level | 99.45% |
| ChatGPT-4 + Prompted Reading Level | 99.50% |
