# Supplementary material for: Evaluating the Readability of Pediatric Neurocutaneous Syndromes–Related Patient Education Material Created by a Custom GPT With Retrieval Augmentation
Source: JMIR Dermatol. 2025 Jul 16;8:e59054. doi: 10.2196/59054 (PMC12286582; doi:10.2196/59054)

Average readability of the GPT Assistant and ChatGPT-4 with and without prompting reading level. An ANOVA test was used to compare differences in reliability of responses (p>0.001)


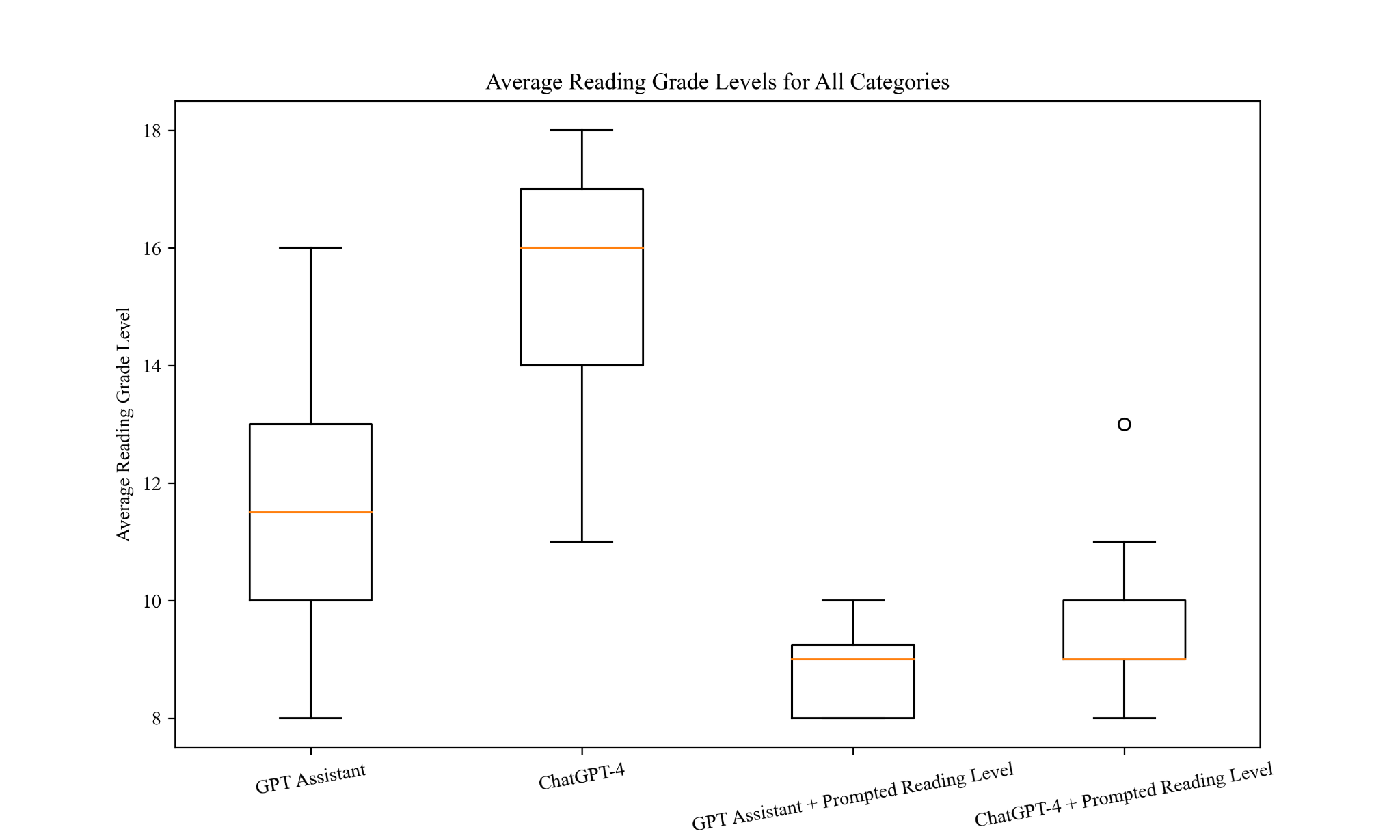

Supplement: Multimedia Appendix 4 [file derma-v8-e59054-s004.docx]
